# Tests of search image and learning in the wild: Insights from sexual conflict in damselflies

**DOI:** 10.1002/ece3.7335

**Published:** 2021-03-17

**Authors:** Silvana Piersanti, Gianandrea Salerno, Viviana Di Pietro, Leonardo Giontella, Manuela Rebora, Albyn Jones, Ola M. Fincke

**Affiliations:** ^1^ Dipartimento di Chimica, Biologia, e Biotecnologie University of Perugia Perugia Italy; ^2^ Dipartimento di Scienze Agrarie, Alimentari e Ambientali University of Perugia Perugia Italy; ^3^ Department of Life Science and Systemtics University of Torino Torino Italy; ^4^ Department of Mathematics Reed College Portland OR USA; ^5^ Department of Biology University of Oklahoma Norman OK USA

**Keywords:** crypsis, intersexual behavior, *Ischnura elegans*, mate recognition, negative frequency‐dependent selection, Odonata, sexual mimicry, visual perception

## Abstract

Search image formation, a proximal mechanism to maintain genetic polymorphisms by negative frequency‐dependent selection, has rarely been tested under natural conditions. Females of many nonterritorial damselflies resemble either conspecific males or background vegetation. Mate‐searching males are assumed to form search images of the majority female type, sexually harassing it at rates higher than expected from its frequency, thus selectively favoring the less common morph. We tested this and how morph coloration and behavior influenced male perception and intersexual encounters by following marked *Ischnura elegans* and noting their reactions to conspecifics. Contrary to search image formation and associative learning hypotheses, although males encountered the minority, male‐like morph more often, sexual harassment and clutch size were similar for both morphs. Prior mating attempts or copula with morphs did not affect a male's subsequent reaction to them; males rarely attempted matings with immature females or males. Females mated early in the day, reducing the opportunity for males to learn their identity beforehand. Once encountered, the male‐like morph was more readily noticed by males than the alternative morph, which once noticed was more likely to receive mating attempts. Flexible behavior gave morphs considerable control over their apparency to males, influencing intersexual encounters. Results suggested a more subtle proximal mechanism than male learning maintains these color polymorphisms and call for inferences of learning to be validated by behavior of wild receivers and their signalers.

## Graphical abstract

In a damselfly with color polymorphic females, mate‐searching males are commonly assumed to form a search image for the majority female morph, which results in disproportionate sexual harassment on it, favoring the minority morph. The resulting negative frequency‐dependent selection should maintain both morphs in the population. By following marked individuals and noting their reactions to conspecifics, we tested this and the role of female influence on the frequency and outcome of male encounters with potential mates. Contrary to search image and associative learning hypotheses, harassment and clutch size were similar for both morphs. Prior experience with either morph did not affect a male's subsequent behavior toward it. Females mated early in the day, reducing the opportunity for males to learn. The male‐like morph was more readily noticed by males, but the alternative morph was more likely to receive mating attempts. Flexible behavior gave morphs considerable control over their apparency to males, influencing intersexual encounters. Results suggested a more subtle proximal mechanism than male learning maintains these color polymorphisms.

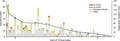

## 1 INTRODUCTION

A major question in evolutionary biology is how natural selection maintains genetic variation in populations (Lewontin, 1974). One commonly cited proximal mechanism for maintaining multiple genotypes is negative frequency‐dependent selection by search image formation (Tinbergen, 1960, reviewed by Punzalan et al. 2005), here defined as a short‐term, perceptual bias in cueing to a given phenotype while ignoring alternative ones. For example, genetic color polymorphisms in cryptic prey species are thought to evolve because they confuse visual predators (e.g., Bond, 2007; Greenwood, 1986; Skelhorn et al. 2011). In turn, searchers are expected to learn to detect the most abundant prey type, cuing to it “apostatically,” at a higher per capita rate than expected from its frequency in the population (Clarke, 1969). Thus, the minority prey type, more likely to be overlooked by predators, should increase in frequency until it becomes the majority and loses its selective advantage. Studies of search image formation in field and laboratory experiments with nonvagile prey support the prediction for such learning in birds (e.g., Allen & Clark, 1968; Bond & Kamil, 2002; Fitzpatrick et al. 2009) and a few invertebrates (e.g. Cross & Jackson, 2010, reviewed by Ishii & Shimada, 2010). Dragonflies and other insects seem physiologically capable of selective attention to items of interest (reviewed by Nityanandra, 2016).

Nevertheless, natural encounter rates of searching predators with vagile, polymorphic prey have yet to be measured due to the difficulty of following predators and their prey in the field. Here, we address two critical questions. Under natural conditions (a) are encounter rates with different morphs high enough to enable search image formation by interested searchers? and (b) how does the appearance and behavior of prey (or other signalers such as mates) affect the rate at which searchers encounter them?

To our knowledge, only one previous study has quantified encounter rates of wild receiver individuals with natural signalers to test for search image formation. Rausher (1978) followed egg‐laying females of the butterfly *Battus philenor* in the field, recording their encounter rates with two species of host plants that differed in leaf shape. The data supported search image for leaf shape. Focal females exhibited greater than expected preference for one of the two plant species, and a few changed their preference based on prior experience, evidence of learned behavior. Nevertheless, because the host plants were not known to be cryptic to the butterfly and it was unclear that its perception changed as the result of experience, Rausher’s (1978) original conclusion of search image formation was later retracted (Papaj & Rausher, 1983). And whereas many wild Hymenoptera pollinators exhibit frequency‐dependent foraging, evidence for disproportionate per capita flower choice is lacking (see Amaya‐Marguez, 2009).

A more apt analogy for vagile, cryptic morphs that challenge searchers is offered by polymorphic females of nonterritorial damselflies whose males must search for potential mates against a visually cluttered background of vegetation. In the family Coenagrionidae in particular, females often exhibit multiple color morphs (reviewed by Fincke et al. 2005). Typically, the andromorph (hereafter, “A‐morph”) is colored and, in some species, patterned like the male, whereas coloration of the heteromorph (“H‐morph,” or gynomorph) differs from the male's, and is more similar to background vegetation (Fincke, 2015; Schultz & Fincke, 2013). For nonterritorial species, these genetic color polymorphisms appear to have evolved in the context of sexual conflict (reviewed by Arnqvist & Rowe, 2005; Chapman, 2006), here manifested as unwanted sexual attention or “sexual harassment” (reviewed by Van Gossum et al. 2008). The assumption that mate‐searching males learn and form a visual search image for the majority morph is commonly used to argue that sexual harassment drives negative frequency‐dependent selection on female color morphs (e.g., Gosden & Svensson, 2009; Svensson et al. 2005; Takahashi et al. 2010). Provided with treatments of airborne chemicals from eight individuals in the laboratory, male damselflies could distinguish different sexes and morph types (Piersanti et al. 2014). In the field, however, males reacted sexually toward female morphs only when they could see them (Rebora et al. 2018a, 2018b). Note that natural selection on female morphs such as that arising from temperature (Svensson et al. 2019) can affect equilibrium morph frequencies but should do so independently of any morph‐specific male visual perceptual bias (Fincke, 2004).

Using the damselfly *Ischnura elegans*, we here test whether natural encounter rates with mature female morphs are high enough to permit search image formation by searching males. Unlike vertebrates, which are logistically difficult to follow in the wild as they search for cryptic prey, marked male and female damselflies can be followed continuously for as long as several hours under natural conditions (Fincke, 2015; Rebora et al. 2018). Thus, we could quantify the rate and kinds of interactions between mate‐searching males and conspecifics, using a receiver's behavior as a reflection of its perception and intent.

Three hypotheses predict that male learning to identify morphs drives negative frequency‐dependent selection on female morphs. First, the Learned Mate Recognition Hypothesis (Fincke, 2004; Miller & Fincke, 1999) explicitly predicts search image formation by mate‐searching males. It posits that males learn from successive contacts with the most abundant female morph to correctly identify or “recognize” that phenotype as a potential mate. Males then perceive and sexually harass the majority morph so aggressively that they depress its relative fitness, thereby favoring the minority morph. For their part, female morphs are expected to assort across the landscape such that harassment costs are equal (Fincke, 2004). Second, in Sherratt’s (2001) analytical Signal Detection Model of Male Mimicry, A‐morphs are assumed to mimic males effectively enough that they are harassed less often than H‐morphs. Once noticed or “detected,” H‐morph females are assumed to always be recognized by males. As the ratio of A‐morphs to males in the population increases, the model results suggested that males learn to recognize them, perhaps by forming a search image, while also making more mistakes in recognizing males. A third hypothesis seems to predict associative learning by positing that a male preferentially attempts to mate with the morph that he mated most recently, while perhaps learning to avoid unsuitable individuals (Takahashi & Watanabe, 2009).

If a male's “encounter” (i.e., being within a detectable distance of an individual, regardless of whether it is noticed) is random, we expected encounter rates of focal males with different female morphs to be frequency‐dependent, with the majority morph (H‐morph in our study population) encountered more often than the minority one (A‐morph). We expected nonrandom encounters if males learn to identify potential mates by association that subsequently leads to search image formation and/or if female behavior influences intersexual encounters. Learning to recognize potential mates presumes that male damselflies have a memory at least as long as the mean encounter interval between two females of the same morph type. Insectary experiments demonstrated that a male's morph preference changed from one day to the next, suggesting that the memory of *Ischnura* and *Enallagma* males persists no more than a day (Takahashi & Watanabe, 2009; Van Gossum et al. 2001a; Xu & Fincke, 2011). If a male learns the identity of female morphs anew each day, we expected that he would (a) have sufficient time to experience different female morphs, a prerequisite for learning, (b) react sexually toward a morph with which he had more experience in recognizing or mating (associative learning, a prerequisite for search image), (c) react sexually toward one morph more often than expected from its encounter frequency while ignoring the alternative morph (search image formation), and possibly (d) avoid mating attempts with inappropriate partners such as immature females (rarely receptive to copula) or males, after previous experience with either (avoidance learning).

We followed focal female morphs to test predictions of search image hypotheses, namely whether in the wild, female morphs are (a) cryptic to males (b) harassed differentially, and (c) assort differentially across the landscape, such that harassment costs are equal. Relevant to these questions and agnostic to male learning, the Signal Apparency Hypothesis defines morph “apparency” as the ease with which male receivers recognize female morphs in the context in which they are found (Schultz & Fincke, 2013). Analogous to perceptual studies of predators and prey (e.g., Endler, 1978; Théry & Gomez, 2010), the hypothesis is based on the visual perception abilities known for coenagrionid damselflies and birds, coupled with the reflectance properties of mature female morphs, males, and background vegetation. For female‐polymorphic *Enallagma* damselflies, the green to brownish H‐morphs exhibit lower chromatic and achromatic (brightness) contrast against background vegetation, compared to the brighter, blue A‐morphs, whose reflectance properties are similar to those of the blue male (Schultz & Fincke, 2013, see Van Gossum et al. 2011, Huang et al. 2014 for similar reflectance properties of *Ischnura*, the sister genus of *Enallagma*). Thus, once encountered, H‐morphs among natural vegetation are expected to be more difficult for a male to notice than the brighter A‐morphs. In contrast, once detected, the male‐like A‐morphs are expected to be relatively more difficult for a male to recognize as a potential mate. Whereas receptive females seeking a mate could behaviorally make themselves conspicuous to males, unreceptive females should minimize their apparency, thus reducing the costs of harassment (Fincke, 2015).

Males might gain additional mating opportunities while females oviposit (e.g., *Enallagma*, Fincke, 1986), when males could identify potential mates from their oviposition behavior alone rather than by relying on color cues. Thus, we also followed egg‐laying females. Because search image formation by harassing males is expected to result in greater fitness for the minority morph, we also counted eggs laid by females directly after completing copula in the field.

## 2 MATERIAL AND METHODS

*Ischnura elegans* is a common European damselfly whose males engage in scramble competition for mates around ponds and calm lake shores. In our long‐term study population that breeds on ponds of the fish hatchery adjacent to Lago Trasimeno, near Perugia Italy (43°05′12.8″N 12°09′05.4″E), males search for receptive females in vegetation around the ponds and are also found in copula in surrounding fields. Our study was done on sunny days during three weeks of August 2017 and over three‐week periods in September of both 2017 and 2018.

**FIGURE 1:**
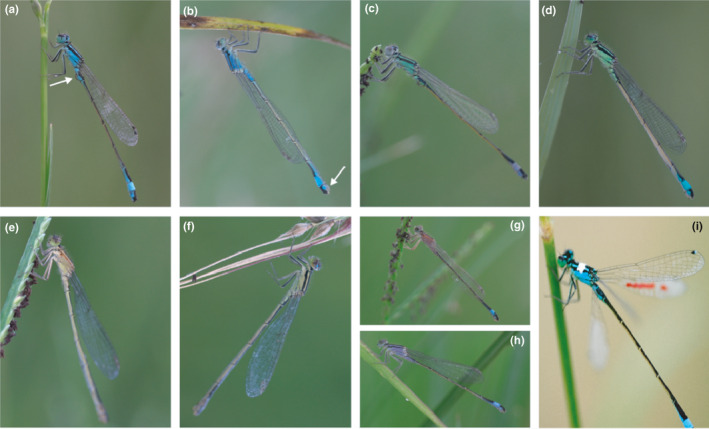
*Ischnura elegans* (a) Blue male, arrow shows penis (b) BA‐morph, arrow shows ovipositor (c) Green male, (d) GA‐morph, (e) H1‐morph, (f) H2‐morph, (g) orange immature female, (h) violet immature female, and (i) marked focal male. Photos by V. Di Pietro

All sexually mature *I. elegans* A‐morphs have a bright blue band on the lower abdomen like the mature male (Figure 1a). A mimic of mature males, the blue andromorph (BA‐morph), has a blue thorax (Figure 1b) whereas the immature male with its bright green thorax (Figure 1c) is mimicked by a green andromorph (GA‐morph, Figure 1d). The latter morph is absent in most European populations of *I. elegans* (but see Longfield, 1949). Lacking the abdominal blue band are two kinds of H‐morphs: *infuscans obsoleta* (hereafter, H1) has one thoracic stripe (Figure 1e) whereas *infuscans* (H2, Figure 1f) has two such stripes (Askew 1988). Sexually immature females have a blue abdominal band and developmental coloration that varies between deeper browns and orange (Figure 1g), or pale blue and violet (Figure 1h).

### 2.1 Opportunity for males to encounter receptive females

To understand the probability that mate‐searching males find receptive females over the course of a day, we counted copulating pairs at the ponds, periodically between dawn and 16:00 hr. One observer walked slowly in the water adjacent to shore without disturbing the unmarked pairs, calling out the morph of each to a scribe. In 2017, we saw two copulas before sunrise. Thus, on 14 September 2018, we conducted a predawn census using flashlights along a 20‐m stretch of bank, from 6:12 to 7:46. To avoid disturbing individuals, we marked the location of females, noting morph and behavior.

To estimate expected encounter rates of focal males with female morphs if encounters are random and assess male competition for females (that if high, should favor search image formation), we used censuses of solitary individuals around ponds to calculate female morph frequency and sex ratio. By randomly shuffling through grass and other vegetation while sweeping with an insect net, we collected flushed individuals. This method helped avoid bias that might arise from collecting only sighted individuals along transects because males fly more than females, and both males and A‐morphs typically perch higher on vegetation than H‐morphs (Rebora et al. 2018a, 2018b). We marked individuals with indelible ink on the wing to avoid recounting the same individuals. Sex ratio of mature adults was calculated from the mature females and blue males collected plus an estimate of solitary green males receptive to mating (i.e., the percentage of green males in the copula census). For comparison with other populations of *I. elegans*, in all censuses we noted the two kinds of H‐ and A‐morphs, immature types and males, but we pooled the kinds within each female type for all but one analysis.

### 2.2 Male‐encounter rates and reactions to conspecifics

From shortly after sunrise until noon, we followed blue, sexually mature focal males. On each observation day, the start of observations was measured as minutes after sunrise. In 2017, males were solitary when collected. To quantify more efficiently how previous morph recognition influences a male's subsequent reactions, in 2018 we caught focal males initially in tandem or copula, noting morph identity before separating the pair. These initial state data were considered only as part of an individual's known experience and were excluded from calculations of male‐encounter or recognition rates.

We marked focal individuals with a drop of white correction fluid on the thorax, which enabled us to follow those that flew several meters. We added a unique pattern of colored marks on the wings with indelible markers to avoid confusing a focal individual with others marked previously (Figure 1i). These methods did not appear to damage individuals; many were seen on subsequent days. We released each focal male on a plant stem at mid‐height, noting the start time of observations. Observers stood as still as possible a meter away from a focal individual, a distance that permitted conspecifics to pass by unimpeded. Individuals were followed until observers lost sight of them for more than five minutes, until copulation began, until there were no encounters with conspecifics for 20 min, or after an hour if the focal individual was foraging. If a focal male was resighted after observations ended, we recorded the time and whether or not it was solitary or in tandem but used only the original duration in analyses. Encounter rate is the sum of an individual's total encounters with unique conspecifics divided by observation duration (excluding the duration an individual was lost and of no activity due to clouds obscuring sun). In 2017, two observers watched each focal male, one as observer and recorder, permitting the other to check the identity of encountered individuals more carefully. In 2018, we added a third observer to reduce the chance of losing a focal male. Excluding males lost within a minute of their release with no interactions, we followed 79 focal males (39 in 2017 and 40 in 2018), for a total of 60 hr, an effort of 145 person‐hours.

For each encounter, we noted the conspecific “type” (male, female morph, color type of immature female) by cueing to color and distinguished between A‐morphs and males by noting the presence of a penis on the second abdominal segment (Figure 1a) or an ovipositor (Figure 1b). We recorded any subsequent behavior by the focal male to determine whether he detected the encountered individual and recognized it. We classified a total of nine reactions, only the most extreme of which was used in analyses: (a) “fly‐by,” considered as an ignored encounter when a focal male flew within 10 cm of a conspecific (the distance over which an individual can be detected, Schultz & Fincke, 2013) without otherwise reacting to it (i.e., the male was either uninterested in the individual or did not perceive it), (b) a “hover” from above or behind the individual, (c) “face‐off”—hovering in front of an individual, often while bouncing up and down, (d) chase, which typically followed a face‐off, (e) grab, (f) “hit”—male hits a conspecific from above, sometimes knocking it to the ground, (g) tandem‐attempt—male tries to engage his claspers with mesostigmal plates on the thorax, (h) tandem—male claspers engage with an individual's thorax, and (i) copula—female raises her abdomen to permit her genital opening to engage with a male's penis (wheel formation). “Sexual reactions” (e–i) were those that most accurately differentiated sex and female maturity (Table 1), suggesting that a male recognized a female morph as a potential mate. If directed toward a male or immature female, we interpreted a sexual reaction as a mistake in recognition. Marking interacting conspecifics would have disrupted focal individuals; we ignored the few individuals that remained in view and interacted a second time (none were mature females). We also recorded but do not report for lack of added insights, the 90 encounters of focal males with tandem or copula pairs whose morph identity had also been noted and encounters initiated by immature females and males toward focal males.

**TABLE 1:** The frequency of most extreme reaction by focal males toward conspecific types and by focal H‐ and A‐morphs toward males

Male reactions	Indicative of	Conspecific type					
		H‐morph		A‐morph		Ifem	Male
		MF	FF	MF	FF	MF	MF
Nonsexual							
Fly‐by	Encounter	6	27	3	65	35	162
Hover	Detection	2	52	6	16	24	101
Face‐off	Detection	5	40	9	40	73	309
Chase	Detection	0	1	1	1	4	29
Frequency of total		**0.78**				0.89	0.98
Sexual							
Grab	Recognition	1	1	2	3	5	2
Hit	Recognition	2	8	2	4	7	8
Tandem‐attempt	Recognition	1	12	0	3	1	0
Tandem	Recognition	4	21	4	8	1	4
Attempt copula^a^	Recognition	0	1	0	1	0	0
Frequency of total		**0.22**				**0.11**	**0.02**

Female reactions to males	H‐morph	A‐morph
No reaction	1	15
Perch low in grass	3	6
Sidle^b^	**25**	**1**
Curve abdomen or Wing raise^c^	**11**	**53**
Change perch or fly	**12**	**57**
Face‐off	7	20
Hit	0	4
Break tandem	6	1
Head nodding post‐tandem	1	2

Note

MF: male‐follow study (2017, 2018); FF: female follow (2017). *p* < 0.05, in bold, chi‐square tests for male reactions, for female reactions, see text. A‐morphs showed a trend for no reaction (*p* = 0.07).

^a^ Male jerks tandem individual, stimulating receptive females to copula position; as a sex‐limited behavior, copula was not compared here.

^b^ Turn on stem while remaining vertical (Corbet, 1999).

^c^ The two behaviors often occurred simultaneously so were counted as one.

To visualize the realized landscape of female morphs encountered by focal males, while accounting for differences in male activity and controlling for observation duration, we examined partial correlations between focal male encounters with each female morph and other males.

To assess whether a male's experience with a given conspecific type affected his subsequent behavior with that type, we used an open source Markovian behavioral model (https://github.com/olivierfriard/behatrix) to identify the behavioral transition states of males that had at least two interactions (i.e., one behavioral “transition”) with a given conspecific type. Our expected null for learned morph recognition was that males encountering a given morph only once during observations were as likely to react sexually to it as males encountering that morph a second time. Learning males should exhibit more sexual‐to‐sexual transitions than sexual‐to‐nonsexual ones. If males form a search image, not only should sexual‐to‐sexual transitions be higher for the most frequently encountered morph, but morph recognition ratios should be significantly higher than morph encounter ratios. If males learned to avoid inappropriate sexual reactions toward immature females or males, we expected more transitions from sexual‐to‐nonsexual responses (including fly‐bys), than transitions from nonsexual‐to‐sexual responses.

### 2.3 Effects of female coloration and behavior on male encounters with potential mates

Beginning shortly after sunrise, in 2017 we followed 31 H‐ and 31 A‐morphs, most caught as solitary females, for a total of 27.6 hr of observation. Methods were similar to those of the male‐follow study except that females were released initially in a low position on a grass stem, and we recorded perch changes and height relative to surrounding vegetation (i.e., low, mid, or high). Sexual reactions from males were scored as sexual harassment except for tandems that remained unbroken by the end of an observation, and copulations, which require a female's cooperation (reviewed by Fincke, 1997). The most realistic indicator of male competition for potential mates is the operational sex ratio (OSR) of sexually receptive adults. We calculated OSR using only the proportion of copulas by focal females initially captured when solitary; all blue males and 12% green males (Table 2) were assumed to be sexually receptive. Controlling for initiation time and duration of observations, focal female encounters with blue males were expected to be similar to those of the blue focal males with females multiplied by 2.34, the estimated ratio of blue males to mature females in the population (Table 2), a proxy for male competition for mates.

**TABLE 2:** Female and male types collected solo and found in copula by year

Year	Type	Solo	Copula	Mature M:F
2017	Immature females	259	10	
	H1‐morph	174	129	
	H2 morph	166	88	
	H‐morph	**0.71**	0.63*	
	BA‐morph	111	88	2.50:1*
	GA‐morph	24	39	
	A‐morph	**0.30**	**0.36**	
	B‐male	1,113	298	
	G‐male	469	56	
2018	Immature females	128	10	
	H1‐morph	61	298	
	H2 morph	34	166	
	H‐morph	**0.64**	**0.69**	
	BA‐morph	36	185	**1** **.38:1**
	GA‐morph	20	21	
	A‐morph	**0.35**	**0.31**	
	B‐male	205	630	
	G‐male	218	50	

Note

B refers to blue, G to green. M = males, F = females. Morph frequency and sex ratio in bold. ^*^ *p* < 0.05, chi‐square tests for solo versus copula within morph types, and sex ratio between years.

To understand the availability of receptive females across the day, we included previously unpublished data from a 2016 female‐follow study conducted later in the day at the same site (Rebora et al. 2018a). Then, most focal females were captured in copula because solo females were harder to find. To determine whether morphs differed in their probability of being encountered but ignored, detected, or recognized by males, as inferred from their reactions to females (Table 1), we used pooled focal female data across years along with reactions from focal males that encountered a given female only once (i.e., independent male reactions), to increase statistical power. The predicted probability of detection and recognition depends on the reflectance properties of males, females, and their background vegetation. Thoracic reflectance of green males is similar to that of the H2‐morph (Henze et al. 2019) but lacking the reflectance data for green A‐morphs and background vegetation, we analyzed GA‐morphs separately. Doing so enables comparisons with most other *I. elegans* populations, which lack GA‐morphs, and *Enallagma* species whose reflectance data underlie the Signal Apparency Hypothesis.

To determine whether males also find mates among egg‐laying females, in 2018, on three days between 15:00 and 18:00 hr, we followed 48 females, 21 A‐morphs (of which six were GA‐morphs) and 27 H‐morphs (of which nine were H2‐morphs) that were laying eggs in mats of aquatic vegetation close to shore. Because egg‐laying females were widely spaced and each remained in a small area for long periods, we followed unmarked females for about 10 min without disturbing them, noting whether they were in sun or shade. We counted all males within a 30‐cm radius around the female and noted any intersexual interactions.

To measure daily fitness of female morphs, starting at 12:30, we held copula pairs in netted cages until they broke tandem naturally, around 15:30. We then put each female individually into a tube with a strip of moistened filter paper as an oviposition substrate. Filter paper strips were replaced daily for three days, until all females had died. Eggs were counted after strips had dried.

### 2.4 Statistical analyses

To assess the prediction that males encounter the majority H‐morphs more frequently than minority A‐morphs, we used a generalized linear mixed model (GLMM) with a Poisson response, male ID as a random effect, morph type as the explanatory variable, and total encounters as the response variable, controlling for observation duration.

To determine the ability of focal males to recognize potential mates, we used a GLMM with a binomial response using the logit link, male ID as a random effect, conspecific type (H‐morph, A‐morph, immature female, male) as the explanatory variable, and sexual versus nonsexual reaction as the response variable. To assess whether a male's experience with a conspecific type affected subsequent behaviors toward that type, we used likelihood ratio tests. For tests of single proportions, we used Clopper–Pearson exact tests.

To test whether males perceived H‐ and A‐morphs differently, we used a generalized linear model (GLM) with a binomial response using logit link, morph type as the explanatory variable, and detection versus recognition as the response variable. We made two comparisons: (a) detection (sexual + nonsexual response) versus encounter only (fly‐by) and (b) recognition (sexual response) versus detection only (nonsexual response). To test whether focal H‐ and A‐morphs received different rates of male harassment, we used a GLM with a binomial response using logit link, morph type as the explanatory variable, and sexual (excluding copula) and nonsexual reactions as response variables, controlling for observation duration. To determine whether focal H‐ and A‐morphs differed their responses to males, we used a GLMM with a binomial response using logit link, female ID as a random effect, morph type as the explanatory variable, and behavior type as the response variable.

To test for unexpected deviations in the intersexual encounters of focal males and focal females, we used a GLM model with a negative binomial distribution with sex, minutes of observation and start time in minutes after sunrise as explanatory variables, and total encounters as the response variable (MASS package, Venables & Ripley, 2002). All above models were done using R (R Core Team, 2018); Z scores refer to Wald tests. Unless noted, means are reported ± standard error.

## 3 RESULTS

### 3.1 Estimates of morph frequency, sex ratio, and availability of potential mates

To serve as a baseline for null expectations of encounter rates of focal individuals with conspecifics, we pooled data from different years. We estimated the population frequency of H‐morphs as 70%, at a ratio of 2.3:1 (H: A, Table 2). Morph frequency of solitary females was similar between 2017 and 2018 ($\chi_{1}^{2}$ = 2.6, *p* = 0.11). Of the total 1,021 solitary females collected, 387 (38%) were immature. Roughly 2/3 of the 2,005 solitary males collected were blue.

**FIGURE 2:**
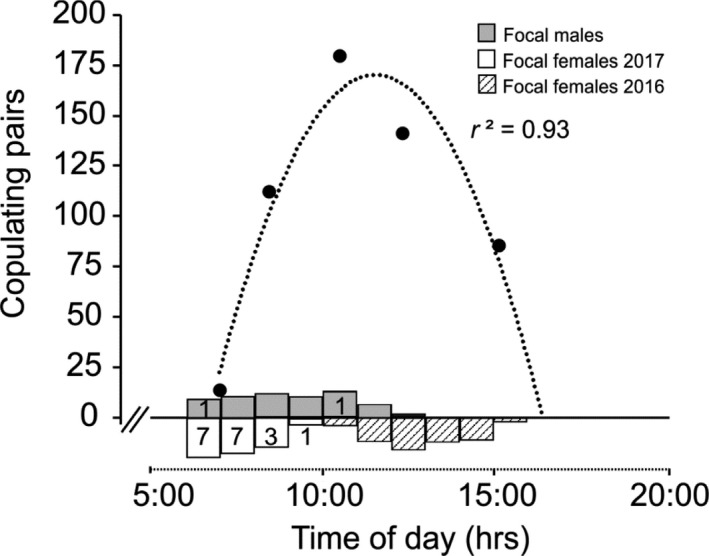
Copula census data (large dots) and initiation times (boxes) of male and female focal observations, showing the pattern of *Ischnura elegans* reproduction of across the day, and the correspondence between initiation of focal observations and copulas. The dotted curve is best fit to quadratic (*y* = −7.3 *x* ^2^ + 167.6 *x* – 793.9, *r* ^2^ = 0.93). Box depth indicates the number (*y*‐axis scale, positive for both males and females) of observations started in each time span. Numbers indicate copulas by focal individuals collected when solitary (i.e., of 47 males, 59 females in 2017, and 39 females in 2016)

In the census of individuals copulating at the water's edge, pairs increased over the morning, reaching a peak between 10 and 11:30, then declined slowly (Figure 2). Most couples had separated by 15:30 hr, when many individuals on the banks adjacent to ponds were solitary females. In the predawn census, we saw females and males crawling to the top of grass stems in the dark. Females remained perched whereas males began to fly 10 min prior to sunrise at 6:49. The first tandem noted was at 6:51, before the sun rose sufficiently to illuminate the pond at 7:23. Three females sighted as solo were in tandem or copula 25, 34, and 113 min later. Of the 10 H‐ and 3 A‐morphs sighted, 9 were in tandem or copula by 7:46. Similarly, across the male and female focal studies, on average, sunrise was at 6:17 and the mean initiation time of the 20 copulas observed was 118 min after sunrise, roughly 8:00 hr (range 6:32–9:30).

### 3.2 Are male encounter rates with female morphs sufficient for mate learning?

**FIGURE 3:**
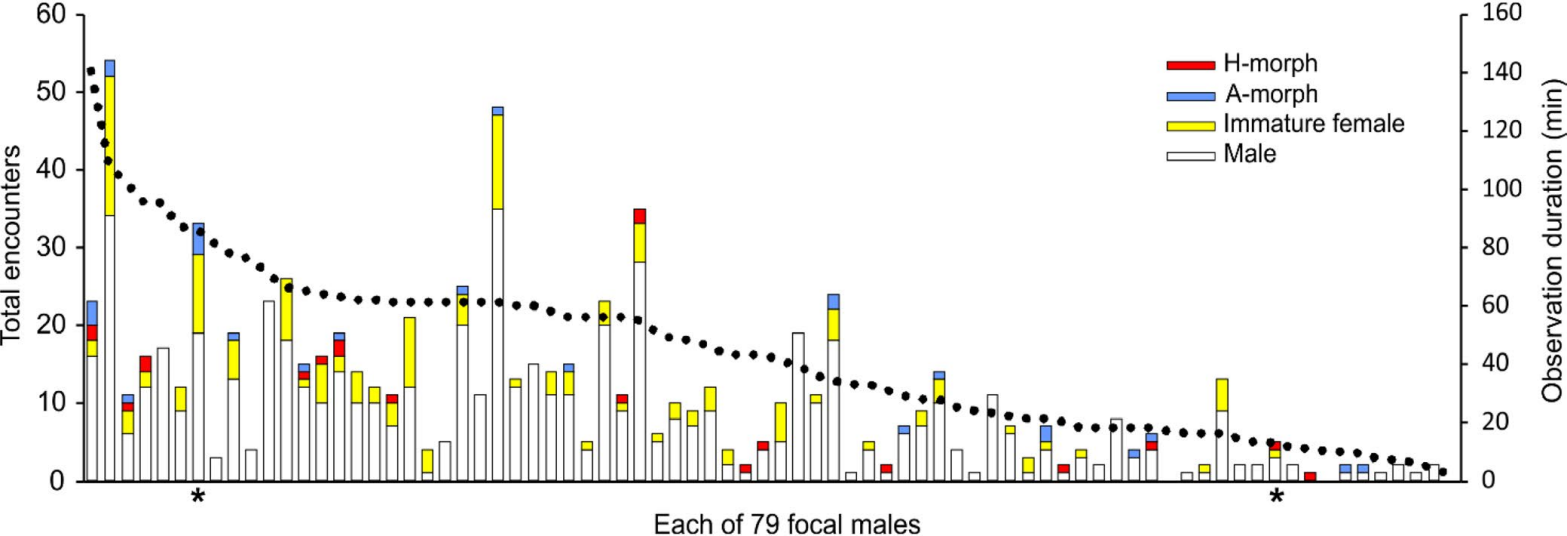
The number of each conspecific type encountered by each of the 79 focal males, in order of observation duration from high to low (black dots, right axis). *male that copulated

**FIGURE 4:**
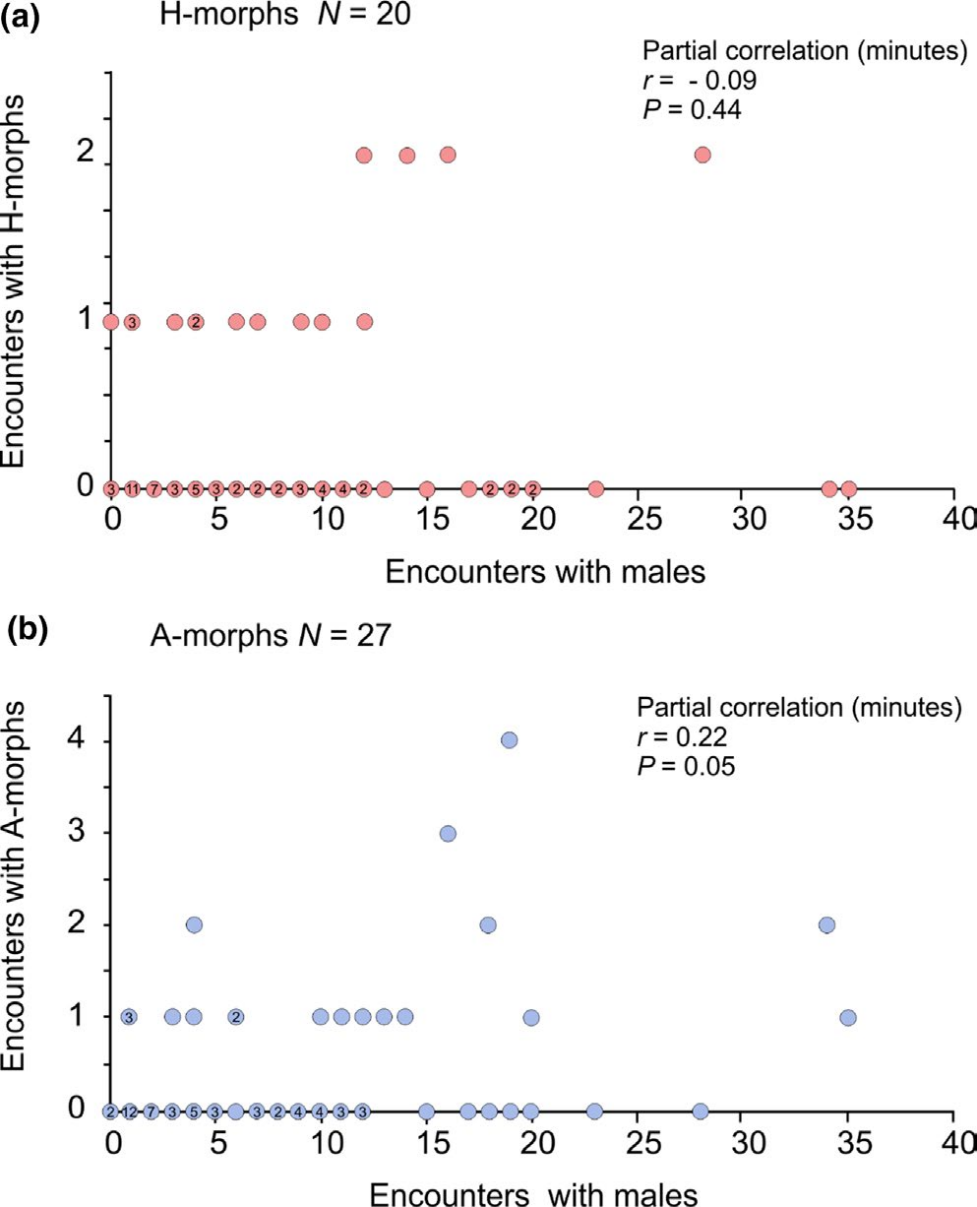
Partial correlation, accounting for observation time, between focal male encounters with other males, and (a) heteromorphs and (b) andromorphs, to visualize the realized landscape of female morphs encountered by focal males, accounting for male activity. Numbers within circles indicate number of focal males represented by the same point

The expected encounter ratio of 2.3 H‐ to A‐morphs, based on morph frequencies (Table 2), was well outside the 95% confidence interval (0.41–1.32) of the estimated observed male encounter ratio of 0.74 H:A (Figure 3, GLMM model). Focal males encountered 47 mature females, 27 A‐morphs at a mean rate of 0.013 ± *SD* 0.037 per min and 20 H‐morphs ($\bar{x}$ = 0.007 ± 0.018 per min). As focal males became more active, encounters with H‐morphs did not change (partial correlation, *r* = −0.09, *p* = 0.445), whereas those with A‐morphs increased (*r* = 0.22, *p* = 0.049, Figure 4). All but two encounters with mature females were initiated by the focal male.

Encounters by focal males with females increased with observation duration (*r* = 0.47, *p* < 0.01). Accounting for observation duration, the earlier the start time, the more likely a male was to encounter a mature female (partial correlation, *r* = −0.39, *p* < 0.001). We followed males for a mean of 42.02 ± 28.4 *SD* min (1–130 min), starting on average 154.10 ± 92.2 *SD* min after sunrise. Males encountered a mean of 10.4 ± 1.17 conspecifics, a rate of about one every three minutes (0.27 ± 0.02/min) and encountered mature females at a mean rate of 0.02 ± 0.0/min, roughly one per hour. In total, focal males encountered 625 males, of which 51% were mature blue, and 197 females, of which only 47 (23%) were mature (Figure 3). Thirty focal males (38%) encountered one or more sexually mature females; 25 (32%) encountered only immature females, and 25 males (32%) never encountered any female during observations. Only two males achieved a copula during observations; another was in tandem when lost, and one lost at 8:49 was seen in copula at 13:15. After an hour, one focal male was captured by a dragonfly.

### 3.3 How well do males recognize conspecifics?

**FIGURE 5:**
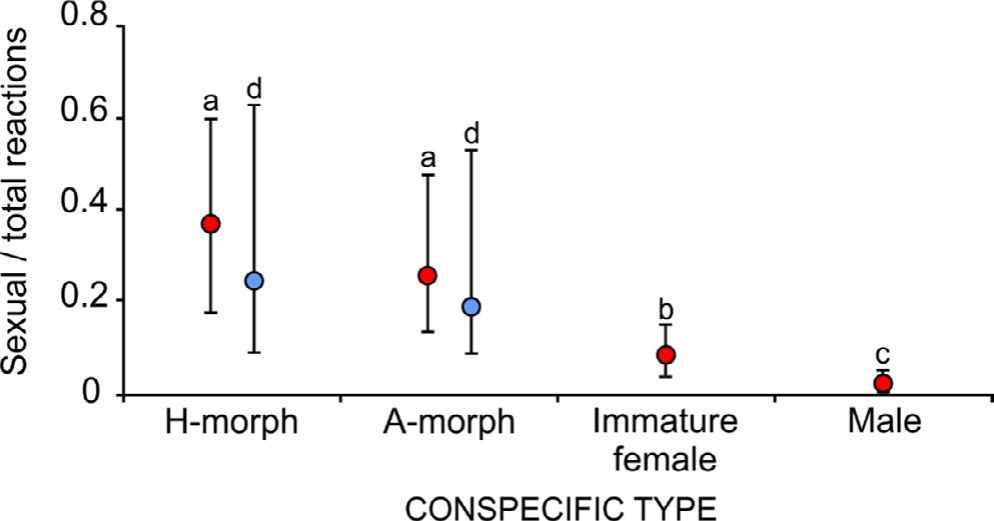
Probability of sexual reactions toward conspecific types. Red dots indicate the probability of sexual reactions ± 95% confidence intervals by focal males toward an encountered type, controlling for male ID (*N* = 20 heteromorphs, 27 andromorphs, 150 immature females, 625 males). Blue dots depict probability of sexual harassment (sexual reactions excluding copulas) ± 95% confidence intervals received by focal females (31 H‐morphs, 31 A‐morphs), controlling for observation duration. Different letters indicate significant differences among types (by color)

Focal males responded differentially to mature females, immature females, and males (Figure 5). The probability that a male responded sexually toward H‐ or A‐morphs was similar (*Z* = 0.806, *p* = 0.42), but was lower toward immature females than either H‐morphs (*Z* = 3.03, *p* = 0.003) or A‐morphs (*Z* = 2.17, *p* = 0.03). Males were more likely to react sexually toward immature females than males (*Z* = 3.75, *p* ≪ 0.01).

### 3.4 Does male learning explain a male's ability to recognize potential mates?

**FIGURE 6:**
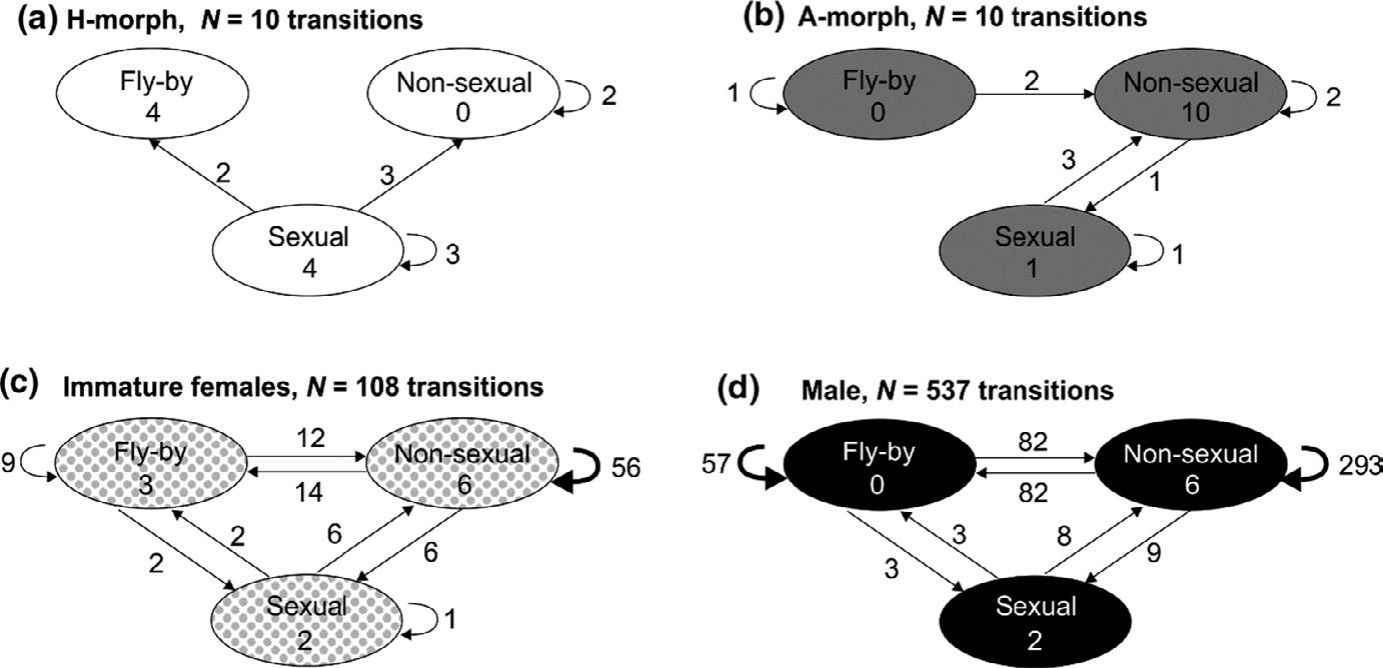
Behavioral transitions (initial and subsequent reaction) of focal males by conspecific type encountered. Ovals depict possible reaction states. Arrows leading from a given reaction indicate the number of times which, on a subsequent encounter with that type, males reacted with a fly‐by (ignored encounter), nonsexual behavior (detection), or sexual behavior (recognized: a, b; mistaken recognition: c, d). Wider arrows indicate statistically significant transitions. Numbers *within* ovals are reactions from males that encountered a given type only once. If males learned to recognize a given morph, sexual‐to‐sexual transitions should be greater than sexual‐to‐fly‐by plus nonsexual, or than single encounters with the morph. If males then formed a search image for the most encountered morph, the proportion of sexual‐to‐sexual transitions in b) should be greater than in a). If males learned to avoid immature females and/or males as mates, transitions from sexual‐to‐fly‐by plus nonsexual should be greater than those from nonsexual plus fly‐by to sexual. None of the above learning predictions were met; see text

Sexual reactions by focal males toward a given conspecific type were unaffected by previous encounters with it. First, males that encountered any mature female only once were as likely to react sexually to it (5/19) as males encountering mature females twice (4/20, *p* = 1.0; Figure 6a,b). Males reacting sexually to either an H‐morph or an A‐morph were as likely to subsequently react nonsexually to the given morph as sexually (5/8 vs. 3/8, *p* = 0.73 Figure 6a; 3/4 vs. 1/4, *p* = 0.62; Figure 6b). Males were more likely to react nonsexually than sexually toward immature females (99/108 vs. 9/108) and other males (513/525 vs. 12/525, *p* ≪ 0.01 for each).

Second, males that reacted sexually toward immature females (Figure 6c) or males (Figure 6d) were not more likely to subsequently react nonsexually to them. Specifically, if males learned from mistaken identity, we expected transitions from sexual‐to‐nonsexual responses toward immature females (1/9) to occur more often than vice versa (8/99). But those ratios were similar (*p* = 0.76), as was the case for the same transitions involving males (0/11 vs. 12/514, *p* = 0.48).

Third, in 2018, of the 40 focal males collected in copula early in the day, only 16 (40%) encountered a second female during observations. Of the nine males encountering the same morph as in their previous copula, only three reacted sexually to it; six males reacted nonsexually to it ($\chi_{1}^{2}$ with Yates continuity correction, *p* = 0.35). Of the seven males encountering a morph different from their earlier partner, all seven reacted sexually to the newly experienced morph.

Finally, counter to explicit predictions of search image formation by focal males, the morph recognition ratio of 1:1 A:H (Figure 5) was not significantly higher than the 1:35 A:H morph encounter ratio (inverse of 0.74 H:A above). Nor was the sexual to sexual behavioral transitions of the encounter‐majority A‐morphs greater than those of H‐morphs (1/4 vs. 3/8, Figure 6, b and a, respectively, *p* = 1.0).

### 3.5 Focal female study: effects of female coloration and behavior on male encounters

Focal female observations began earlier (*t*_139_ = −5.70, *p* < 0.001) and were shorter (*t*_139_ = −2.97, *p* = 0.003) than those of focal males. Starting on average 82.9 ± 54.0 *SD* minutes after sunrise (range, −1 to 182), females were followed for a mean of 27.8 ± 27.9 *SD* min. ($\bar{x}$ = 23.4 ± 3.7 min, 31 H‐morphs; $\bar{x}$ = 36.4 ± 6.4 min, 31 A‐morphs).

Of the 253 focal female encounters, 98% were with males (66% with blue ones); all but two were initiated by the males. Focal A‐morphs encountered more males than H‐morphs (pooled data, *F*_2,89_ = 4.78, *p* = 0.031). Of the 59 focal females collected as solitary, 18 (30%) copulated (seven with green males), resulting in an operational sex ratio (OSR) of 8.2 (M:F). Marking focal females with white‐out did not seem to make them more detectable to males. In 2017, focal females had a greater probability of receiving a male fly‐by relative to being detected (i.e., nonsexual plus sexual response) than females in 2016, which lacked white marks (logistic regression, Wald χ^2^ = 7.62, *p* = 0.006).

Encounters of focal females with blue males were more frequent than encounters of focal males with mature females (*Z* = −6.73, *p* ≪ 0.01), after accounting for start time (*Z* = −2.62, *p* < 0.01) and observation duration (*Z* = 6.34, *p* ≪ 0.01). The estimated encounters with blue males per focal female were 4.47 (95% confidence interval 2.94, 7.10), greater than the expected 2.34 ratio (*p* < 0.01) of blue males: mature females in the population (Table 1).

**FIGURE 7:**
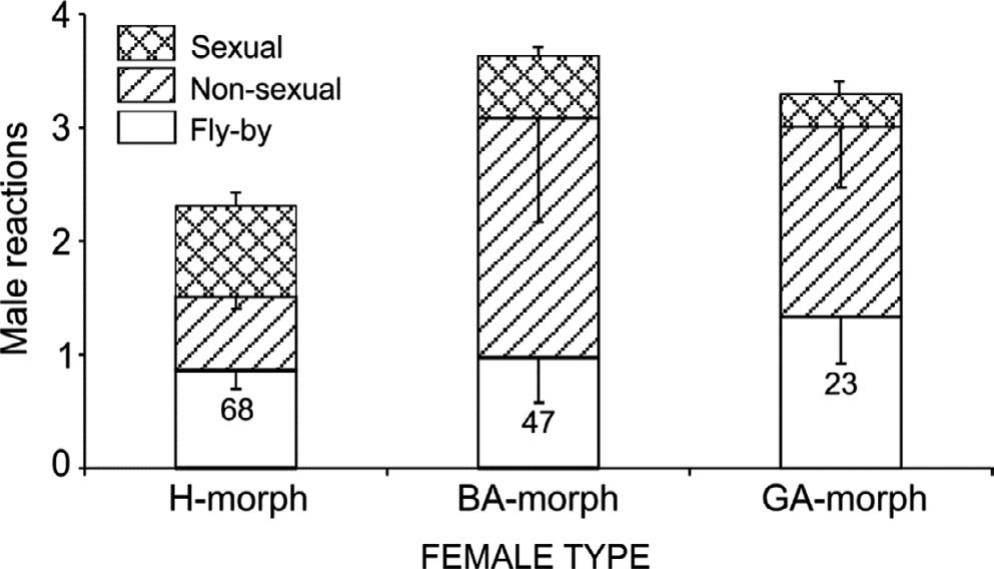
Means ± *SE* of behavioral proxies for male perception of heteromorphs, blue andromorphs, and green A‐morphs. Fly‐by is an ignored encounter, nonsexual reaction indicates that a male detected the female, whereas a sexual reaction indicates that having detected a female, the male recognized it as a potential mate. Fly‐by + Nonsexual + Sexual = total encounters, Nonsexual + Sexual = total detections, and Sexual reactions = total recognitions. The proportion of total encounters that were detections was significantly greater for BA‐morphs than H‐morphs, whereas recognitions were a greater proportion of detections for H‐morphs than A‐morphs (see text). Numbers are sample sizes. Because GA‐morph reflectance properties were unknown, it was not pooled with BA‐morph

Given an encounter with a male, A‐morphs were more likely to be detected (receive nonsexual or sexual responses) relative to H‐morphs (*Z* = −2.06, *p* = 0.039, Figure 7). If detected, focal H‐morphs were more likely than A‐morphs to be recognized (receive a sexual response) by a male (*Z* = 5.14, *p* ≪ 0.001). Green A‐morphs were more likely than H‐morphs to be detected by males (*Z* = −2.16, *p* = 0.03). If detected, they were as likely as BA‐morphs to be recognized as a potential mate (*Z* = −0.76, *p* = 0.45).

Sexual harassment from males was similar toward focal H‐ and A‐morphs (*Z* = 0.46, *p* = 0.64; Figure 5). However, A‐morphs received significantly more nonsexual responses from males than H‐morphs (*Z* = −2.30, *p* = 0.02). Focal A‐morphs also tended to receive more total responses from males, relative to H‐morphs (*Z* = −1.78, *p* = 0.07, 2017 data; *Z* = −0.2.51, *p* = 0.01, pooled data).

A‐ and H‐morphs performed similar behaviors but used some with different frequencies (Table 1). To avoid detection from a passing male, focal H‐morphs were more likely than A‐morphs to sidle (Corbet, 1999), remaining vertical while moving around a stem away from the male's approach (*Z* = 3.07, *p* = 0.002), whereas A‐morphs tended to remain stationary (*Z* = −1.8, *p* = 0.07). Once detected, A‐morphs were more likely than H‐morphs to change perch or fly away (*Z* = −2.96, *p* = 0.003). Two H‐morphs curled their abdomens up over their thorax, preventing the male from achieving tandem. On three of four occasions, A‐ and H‐morphs were seen to laterally nod their heads back and forth for up to several minutes before males broke tandem. Five of the 13 (38%) copulas by focal H‐morphs occurred after they perched high on vegetation, where they were taken in tandem within minutes, and proceeded to copulate without resistance. In contrast, after being hit to the ground by a male, two H‐morphs completely buried themselves under the grass layer where they were invisible to us; another proceeded to copula.

The probability of mating did not differ by morph, pooling across years (*χ* ^2^ = 1.32, *N* = 1648, *p* = 0.25, Table 1). In 2017, the probability of mating for H‐morphs was lower than expected based on their frequency in the population (*χ* ^2^ = 6.62, *N* = 819, *p* = 0.01), but in 2018, there was little difference between morphs (*χ* ^2^ = 1.9, *N* = 829, *p* = 0.27).

Males did not harass egg‐laying females, regardless of morph type. The number of males within 15 cm of each morph was similar (*F*_1,45_ = 2.30, *p* = 0.14), as were morph encounters with males (*F*_1,45_ = 0.05, *p* = 0.82), accounting for observation time (A‐morphs: $\bar{x}$ = 10.0 ± 0.13 min; H‐morphs: 8.96 ± 0.51 min). Only four females (2 H‐morphs, 2 A‐morphs) encountered a male; none were harassed or changed their position. The most extreme was a face‐off, the remainder, hovers. Encounters with males did not differ between females in sun or shade (*F*_1,46_ = 0.07, *p* = 0.79). An A‐morph in copula on shore head‐nodded before being released by her mate.

Female morphs allowed to oviposit after copula differed little in clutch size ($\bar{x}$ = 260.50 ± 16.20 eggs, range 44–538, *N* = 40 H‐morphs; $\bar{x}$ = 217.81 ± 22.44 eggs, range 33–390, *N* = 19 A‐morphs, *F*_1,57_ = 2.27, *p* = 0.14). Clutch size did not differ among all four types (H1,H2,BA, GA, *F*_3,55_ = 1.58, *p* = 0.20). Most eggs (96% of total) were laid on the day of capture. All females had died by day 3. Excluded from analysis were two anomalous A‐morphs laying only four and ten eggs on day 3.

## 4 DISCUSSION

Our study offers the first test of search image formation by animals under natural conditions that search for vagile polymorphic prey or mates. Results failed to support male search image formation or associative learning, both claimed to be proximate mechanisms resulting in unequal sexual harassment of female morphs, thus driving negative frequency‐dependent selection on color polymorphic females. Rather, females’ morph‐specific appearance coupled with flexible behavior best explained nonrandom male‐encounter rates with potential mates and the equal probability of morph harassment and clutch size that we found.

### 4.1 What we learned by following males in the wild

Focal males rarely encountered more than a single mature female during observations (Figure 3) but were able to correctly differentiate between potential mates and inappropriate conspecifics (Figure 5), an ability that did not depend on a male's prior experience with females on a given day. Males did not exhibit associate learning for either morph, a prerequisite for search image formation. Although their probability of encountering the minority A‐morph was 1.35 times that for H‐morphs (Figure 4), males failed to recognize A‐morphs disproportionally more often than H‐morphs as expected for search image formation (Figure 6a,b). And despite relatively high A‐morph to male‐encounter frequency, males rarely mistook males for females (Figure 5). Thus, results contradicted both the Learned Mate Recognition Hypothesis (Fincke, 2004) and the Signal Detection Model of Male Mimicry (Sherratt, 2001). After being captured in copula in early morning, the subsequent behavior of focal males refuted associative learning and frequency‐dependent harassment (Takahashi et al. 2010). Of those males that encountered a second female, 81% reacted sexually to a morph different from its former mate, contra Takahashi and Watanabe (2009) and single‐encounter search image formation (e.g., Jackson & Li, 2004). Finally, males did not learn from mistaken identity (Figure 6c,d).

Cognitive constraints may preclude learning female identity by mature *Ischnura* males as seems to be the case for *Enallagma* males, which learn to distinguish between mature males and females as immature adults (Fincke et al. 2007, for *Ischnura* males see, also Takahashi & Watanabe, 2011, Sánchez‐Guillén et al. 2013). Free‐flying male *E. hageni* readily responded sexually to live females painted pink, a color that they never before experienced (Xu et al. 2014). That study identified a male's decision rule for female identity (“if green, then female, if blue, then focus on pattern”), which males apparently learned as immatures. Adding to a male's cognitive challenge in our study population are two types of A‐morphs, which should make *I. elegans* females more confusing to males than in areas lacking A‐morph variants.

### 4.2 What we learned by following female signalers in the wild

Despite results of the behavioral transitions being opposite in direction of those expected with learning (Figure 6), following more males in early morning would make our conclusions more robust. However, results from focal females reinforce our conclusion that the low encounters of males with female morphs were a consequence of high male competition for mates. Although we followed males longer than females, for both sexes intersexual encounters increased with observation duration but decreased with time after sunrise. Accounting for those effects, the estimated number of focal female encounters with blue males was 4.5, significantly higher than the 2.5 expected (i.e., blue M:F for 2017, Table 2). Only a third of focal females were sexually receptive, producing an OSR of 8:1. Mean initiation time of copulas (90% by focal females) was roughly 8:00 hr, further reducing a male's opportunity to encounter even one potential mate before females stopped mating for the day (Figure 2). Indeed, some tandems formed in the dark when males presumably could not discern morph coloration. *Ischnura elegans* couples can remain in the copula position for as long as seven hours, a male mate‐guarding strategy (Miller, 1987). Other *Ischnura* sp. have similarly restricted mating periods coupled with long matings (e.g., Cordero, 1992; Takahashi & Watanabe, 2009). Males did not harass focal egg‐laying females, similar to *Ischnura verticalis* (Fincke, 1987, but see Takahashi & Watanabe, 2009 for *I. senagalensis* males that harass such females with no prospect of mating). Lifetime mating success of wild *Ischnura* (Cordero et al. 1998), and *Enallagma* (Fincke, 1994) whose males copulate for a shorter time and do mate with egg‐laying females, indicated that roughly half of males failed to mate even once in their lives. Thus, mate‐searching males might not get another chance if they ignore any mature female morph as a potential mate.

Our results best supported the Signal Apparency Hypothesis (Schultz & Fincke 2013; Fincke, 2015), coupled with the prediction of differential morph assortment (Fincke, 2004). Morph‐specific differences in the probability of being detected and recognized by males (Figure 7), coupled with flexible morph behavior (Table 1, see also Van Gossum et al. 2001b), gave wild females considerable control over their apparency to males and hence, over the frequency and outcome of intersexual encounters. The male‐like minority A‐morph was the majority morph encountered (Figure 4), apparently due to its habit of perching relatively higher on vegetation where males are typically found. Although H‐morphs typically perch lower in the vegetation than either A‐morphs or males (Bots et al. 2015; Fincke, 2015; Rebora et al. 2018a, 2018b; Van Gossum et al. 2001b), focal H‐morphs readily found mates when perched high. Their cryptic coloration in concert with sidling to the opposite side of a grass stem enabled H‐morphs to elude passing males. Importantly, both morphs could break unwanted tandems and used head‐nods, an apparent signal to males of nonreceptivity, after which males broke tandem (see also Forbes et al.1995).

Counter to both the Learned Mate Recognition Hypothesis (Fincke, 2004) and the Signal Detection Model of Male Mimicry (Sherratt 2001), H‐ and A‐morphs had equal probabilities of being harassed (Figure 5). Both hypotheses predicted that the minority‐encountered heteromorphs should be harassed less than the majority‐encountered andromorphs. However, absent male learning of morph identity, this result is best explained by female morphs’ differential assortment across the male‐encounter landscape (Figure 4, see also Forbes et al. 1995). And given their higher rate of nonsexual interactions, A‐morphs were actually disturbed more often than H‐morphs (see also Fincke, 2015; Rebora et al. 2018a). Counter to the expectation that negative frequency‐dependent selection should selectively favor the minority‐encountered H‐morph, daily clutch size of morphs differed little (but see Gosden & Svensson, 2009; Sánchez‐Guillén et al. 2017, using different methods).

The only focal individual that died during observations was a male caught by a dragonfly. Visual predators such as dragonflies and birds should notice the more conspicuous males and A‐morphs more readily (Schultz & Fincke, 2013). Any such bias favoring H‐morphs might help restore chance disruptions of equilibrium morph frequencies or help offset andromorph‐specific effects favored by natural selection (e.g., Sánchez‐Guillén et al. 2017; Svensson et al. 2019). Nevertheless, balancing selection from trade‐offs between predation and sexual harassment (Robertson, 1985) is an unlikely proximal mechanism for maintaining the color morphs, given similar morph lifespans (Cordero Rivera & André, 1999; Fincke, 1994) and perhaps a greater predation threat from nonvisual predators (e.g., Fincke, 1994; Palacino‐Rodríguez et. al., 2020). The heteromorph majority of roughly 70% appeared fairly stable (e.g., between 2012 and 2013, 70.2% of 517 emerging females collected as larvae were H‐morphs, S. Piersanti, unpublished data), suggesting that a more subtle proximal mechanism maintains these polymorphisms than previously envisioned.

## 5 CONCLUSIONS

*Ischnura* damselflies are often used to illustrate a general mechanism by which polymorphisms are maintained by negative frequency‐dependent selection and to model the effects such selection more broadly (e.g., Iserbty et al. 2013; Takahashi et al. 2014; Verzijden et al. 2012). Like models of predators cuing to polymorphic prey, those studies assume that the proximal mechanism driving selection on morph females is search image formation by sexually harassing males. Evidence that mature *Ischnura* males learn to recognize potential mates depends on preference changes of caged males under low sex ratio and high morph density (Van Gossum et al. 2001a) and those of wild males presented with a choice of dead morphs early and late in the day (Takahashi & Watanabe, 2009). Despite a paucity of evidence, Le Rouzie et al. (2015) concluded from their model, that “the inferred decline in fitness of female morphs are indeed caused by apostatic selection due to mating harassment as any given morph becomes more common in the population.” The behavior of wild male and female *I. elegans* explicitly contradicts such conclusions.

Our results call for caution when inferring learned preferences from laboratory or insectary studies, particularly for relatively short‐lived insects (reviewed by Dion et al., 2019). Promising candidates for testing search image in the wild are butterflies that search for polymorphic mates (Kunte, 2009; Turlure et al. 2016) or host plants (e.g. Dell’Aglio et al., 2016), prey‐searching salticid spiders and parasitoid wasps searching for hosts (reviewed by Ishii & Shimada, 2010). Finally, quantifying the lowest density of virtual prey under which birds form a search image in the laboratory (e.g., Bond, 2007) would offer a more realistic prediction of its possibility in the wild.

## CONFLICT OF INTEREST

No conflict of interest.

## AUTHOR CONTRIBUTION

**Silvana Piersanti :** Conceptualization (supporting); Data curation (supporting); Investigation (lead); Methodology (equal); Resources (lead); Supervision (lead); Visualization (supporting); Writing‐review & editing (supporting). **Gianandra Salerno:** Formal analysis (supporting); Investigation (supporting); Visualization (lead); Writing‐review & editing (supporting). **Vivana Di Pietro :** Data curation (supporting); Investigation (equal); Visualization (equal). **Leonardo Giontella:** Investigation (equal); Resources (equal). **Manuela Rebora:** Investigation (supporting); Methodology (supporting); Resources (equal); Supervision (supporting). **Albyn Jones:** Formal analysis (lead). **Ola M Fincke:** Conceptualization (lead); Data curation (lead); Formal analysis (equal); Investigation (equal); Methodology (lead); Visualization (equal); Writing‐original draft (lead); Writing‐review & editing (lead).

## Data Availability

Data and R codes are in Dryad: https://doi.org/10.5061/dryad.xgxd254fm.
